# Platelet Serotonin (5-HT) Concentration, Platelet Monoamine Oxidase B (MAO-B) Activity and *HTR2A*, *HTR2C*, and *MAOB* Gene Polymorphisms in Asthma

**DOI:** 10.3390/biom13050800

**Published:** 2023-05-08

**Authors:** Marcela Konjevod, Katherina B. Sreter, Sanja Popovic-Grle, Marina Lampalo, Lucija Tudor, Irena Jukic, Gordana Nedic Erjavec, Jasna Bingulac-Popovic, Hana Safic Stanic, Matea Nikolac Perkovic, Jasenka Markeljevic, Miroslav Samarzija, Nela Pivac, Dubravka Svob Strac

**Affiliations:** 1 Rudjer Boskovic Institute, Division of Molecular Medicine, Bijenicka Cesta 54, 10000 Zagreb, Croatia; 2 Department of Clinical Immunology, Pulmonology and Rheumatology, University Hospital Centre “Sestre Milosrdnice”, 10000 Zagreb, Croatia; 3 Clinic for Lung Diseases Jordanovac, University Hospital Centre Zagreb, 10000 Zagreb, Croatia; 4 School of Medicine, University of Zagreb, 10000 Zagreb, Croatia; 5 Croatian Institute of Transfusion Medicine, 10000 Zagreb, Croatia; 6 Faculty of Medicine, Josip Juraj Strossmayer University of Osijek, 31000 Osijek, Croatia; 7 University of Applied Sciences “Hrvatsko Zagorje Krapina”, 49000 Krapina, Croatia

**Keywords:** asthma, platelet MAO-B activity, platelet 5-HT levels, *MAOB*, *5HT2A*, *5HT2C* polymorphisms, haplotype analysis, severity, phenotypes

## Abstract

The complex role of the serotonin system in respiratory function and inflammatory diseases such as asthma is unclear. Our study investigated platelet serotonin (5-HT) levels and platelet monoamine oxidase B (MAO-B) activity, as well as associations with *HTR2A* (rs6314; rs6313)*, HTR2C* (rs3813929; rs518147), and *MAOB* (rs1799836; rs6651806) gene polymorphisms in 120 healthy individuals and 120 asthma patients of different severity and phenotypes. Platelet 5-HT concentration was significantly lower, while platelet MAO-B activity was considerably higher in asthma patients; however, they did not differ between patients with different asthma severity or phenotypes. Only the healthy subjects, but not the asthma patients, carrying the *MAOB* rs1799836 TT genotype had significantly lower platelet MAO-B activity than the C allele carriers. No significant differences in the frequency of the genotypes, alleles, or haplotypes for any of the investigated *HTR2A, HTR2C* and *MAOB* gene polymorphisms have been observed between asthma patients and healthy subjects or between patients with various asthma phenotypes. However, the carriers of the *HTR2C* rs518147 CC genotype or C allele were significantly less frequent in severe asthma patients than in the G allele carriers. Further studies are necessary to elucidate the involvement of the serotonergic system in asthma pathophysiology.

## 1. Introduction

Asthma is a complex chronic inflammatory disease of the airways, characterized by the typical respiratory symptoms of coughing, shortness of breath, wheezing, and chest tightness [1]. It affects around 262 million children and adults worldwide [2] and is associated with increased comorbidity [3] and socioeconomic burden that reduces the quality of daily life [4,5]. Asthma is considered a heterogeneous disease with different underlying pathophysiological mechanisms (endotypes) and various clinical presentations (phenotypes) [6]. Although the exact pathophysiological basis of asthma is unclear, it seems to involve a multifaceted network of interacting genetic, environmental, developmental, immunological, and other factors [7].

The involvement of the serotonergic system in pulmonary function [8], as well as in inflammation and inflammatory diseases, such as asthma, has been reported [9,10]. The activities of cells classically involved in asthma are modulated by serotonin (5-HT), such as the regulation of cell adhesion and, thus, migration and cytokine-chemokine production [11]. In addition, it is known that 5-HT has immunomodulatory effects [12] and is involved in airway inflammation through eosinophil-related processes [10]. Several studies have demonstrated increased levels of 5-HT in asthma patients’ plasma and bronchoalveolar fluid [13,14] or during an asthma attack [15]. Moreover, high levels of 5-HT in the blood have been associated with the intensity and exacerbation of asthma [13]. Studies to date have primarily examined free 5-HT levels in the serum or plasma of asthma patients [13,15]. However, precise determination of 5-HT in plasma is difficult due to low concentrations of circulating 5-HT, with the vast majority present in platelets, and the possibility of activating platelets during measurement [16]. Existing data have suggested that platelets and their release products, such as 5-HT, are essential players in various inflammatory diseases, including asthma [11,17].

Furthermore, platelets are actively involved in most of the main features of asthma [18]. However, thus far, even though it is well known that platelets contain the majority of blood 5-HT and that 5-HT plasma levels may be regulated by the uptake or release of 5-HT by platelets [19,20], there is a lack of published reports focused on clarifying the role of platelet 5-HT levels in asthma and their application to identify new asthma phenotypes [21,22,23]. Therefore, this study aimed to investigate the platelet 5-HT levels in asthma patients and their potential association with severity and various asthma phenotypes.

Another factor that regulates 5-HT levels is the activity of monoamine oxidase (MAO), the enzyme that plays an essential role in 5-HT catabolism [24]. Specifically, 5-HT is mainly metabolized via its oxidative deamination catalyzed by the enzyme MAO [25]. Although the two subtypes of MAO (A and B) are located throughout the brain, only MAO-B is present in the platelets of humans [26]. MAO-A catabolizes primarily 5-HT and, to a lesser extent, norepinephrine, whereas MAO-B preferentially degrades β-phenylethylamine and benzylamine [27]. However, despite differing their preferred substrates, neither MAO-A nor MAO-B possesses absolute substrate specificity, so either can degrade dopamine, tryptamine and tyramine [27].

Moreover, in the absence of one of the MAO isoenzymes, the other can totally or partly compensate by deaminating a certain amount of its unfavored biogenic amine substrates [27,28,29,30]. Additionally, MAO-A and MAO-B can oxidize each other’s favoured substrates in the event of a change in the substrate concentration or enzyme concentration [28,31,32]. Specifically, MAO-B is deemed to metabolize catecholamines in a subsidiary fashion when MAO-A activity is inadequate [30]. Therefore, we hypothesized that platelet MAO-B activity influences the platelet 5-HT concentration. Previous research has indicated that MAO is induced in peripheral chronic inflammatory diseases, whereas MAO inhibitor drugs have anti-inflammatory effects [17]. Compounds involved in oxidative stress, such as H_2_O_2_ and reactive aldehydes, are constantly generated as by-products during oxidative deamination by MAO [33]. The enhanced oxidative stress observed in asthma patients is thought to play a critical role in the pathogenesis of this chronic inflammatory disease [34].

Nonetheless, the precise function of MAO-generated oxidative stress and MAO-related inflammation in asthma is still not fully understood. Moreover, as far as we know, the association of platelet MAO-B activity with asthma has not been investigated. Hence, in addition to platelet 5-HT levels, we have investigated the platelet MAO-B activity in asthma patients with different severity and phenotypes. It has been proposed that MAO-B activity is highly heritable [35]. Several *MAOB* gene variants may influence protein expression and platelet MAO-B activity [27]. Specifically, variations in the coding sequences of genes could alter the amino acid sequence, resulting in a changed, truncated, incomplete or non-functional protein. Individual polymorphisms in the *MAOB* gene may have a minimal functional impact; however, they may be in linkage disequilibrium (LD) with a set of polymorphisms that form a haplotype influencing *MAOB* gene expression or function and consequently altering the MAO-B activity. In our study, we investigated two selected (rs1799836 and rs6651806) intron *MAOB* gene polymorphisms and their haplotype. Although polymorphisms located in intronic regions do not modify the protein sequence, more recent evidence indicates that such variations can create splicing abnormalities, which may influence translation and lead to human disease [36].

The various biological and pathophysiological actions of 5-HT are exerted via different 5-HT receptors. Among them, the 5-HT2 receptor class seems to play an essential role in asthma, as shown by animal and human studies [37,38,39]. The 5-HT receptors are widely distributed throughout the respiratory system’s bronchial, nervous, and vascular structures, with the 5-HT1A, 5-HT2A, 5-HT3 and 5-HT7 receptors mainly involved in the control of human airway function [8,40,41]. It has been shown that 5-HT may activate the 5-HT receptors in various inflammatory cells that participate in the development of asthma [42]. Stimulation of the 5-HTR1A, 5-HTR1B, 5-HTR1E/F, 5-HTR2, 5-HTR3, 5-HTR4 and 5-HTR7 receptor subtypes was shown to mediate the release of inflammatory cytokines, such as IL-6 and CXCL8/IL-8, from bronchial epithelial cells [43]. The 5-HT2 receptors are quite important in asthma, as demonstrated by various animal and human studies [36,37,38]. Kang et al. (2013) discovered that the migration of eosinophils in allergic asthma seems to hinge on 5-HT2A receptor activation [43], while Dürk et al. (2013) showed that 5-HT2 receptors are implicated in platelet function relevant to allergic asthma [14]. Increased 5-HT2A expression has been observed in asthma patients’ peripheral blood mononuclear cells compared to the control group [44,45]. In addition, the contractile effects of 5-HT are most probably mediated through 5-HT2A sites located on pulmonary vascular smooth muscle cells [40]. Further highlighting the importance of the 5-HT2 receptors in allergic airway disease are the studies by Nau et al. (2015) and Flanagan et al. (2019) based on animal models of acute and chronic asthma, respectively [38,46]. Moreover, a study on mice has demonstrated that 5-HT2 receptors are required to control plasma 5-HT levels [47]. In contrast, platelets have been shown to express 5-HT2A and other 5-HT receptors, including 5-HT1A, 5-HT1B, 5-HT3A, and 5-HT4 receptors [48,49,50,51,52]. Hence, in our study, we included the *HTR2A* (rs6314 and rs6313) and *HTR2C* (rs3813929 and rs518147) polymorphisms with the hypothesis that they could influence the expression of 5-HT2A and 5-HT2C receptors in both respiratory system and platelets and might be associated with asthma by influencing the unbound 5-HT levels. According to the available data, *HTR2A* and *HTR2C* gene polymorphisms have not yet been incorporated in previous research on asthma, with *HTR4* polymorphisms being the only 5-HT receptor gene variants associated with asthma so far [41].

The described discrepancies between findings and the scarcity of studies investigating the role of the serotonergic system in asthma emphasize the need for further research. Therefore, this study aimed to investigate platelet 5-HT levels, platelet MAO-B activity, and the potential associations of *HTR2A, HTR2C*, and *MAOB* gene polymorphisms in adult asthma patients and healthy control subjects, as well as in asthma patients of different severity and phenotypes.

## 2. Materials and Methods

### 2.1. Participants

The study enrolled 240 adult subjects (120 asthma patients and 120 healthy control subjects) of both genders and of Croatian origin. Asthma patients were recruited at the Outpatient Department of the Clinic for Lung Diseases Jordanovac, University Hospital Centre Zagreb, Croatia. They were diagnosed and classified according to asthma severity based on the Global Initiative for Asthma (GINA) classification guidelines [1]. The control group consisted of volunteer blood donors recruited at the Croatian Institute for Transfusion Medicine, Zagreb, Croatia. The inclusion and exclusion criteria for enrolment of the subjects in the study have previously been outlined in detail [53]. Data regarding age, gender, body mass index (BMI), and smoking were collected for all subjects. A complete medical history, physical examination, and diagnostic workup were conducted on asthma patients. Skin prick testing (SPT), spirometry with bronchodilation, single-breath diffusing capacity of the lung for carbon monoxide (DLCO) test and fractional exhaled nitric oxide (FeNO) measurement were performed. The forced expiratory volume in one second (FEV_1_), forced vital capacity (FVC), and peak expiratory flow (PEF) were calculated as stated previously [53]. The eosinophil and neutrophil counts were obtained from automated complete blood counts.

In contrast, serum immunoglobulin E (IgE) levels were measured using enzyme-amplified chemiluminescent immunoassays (Immulite^®^ 2000XPi, Siemens Healthcare Diagnostics, Erlangen, Germany), according to the manufacturer’s procedures. As already described [53], asthma patients were subdivided into non-severe (mild-to-moderate) and severe asthma patients [1], as well as into patients with non-allergic and allergic asthma [54], eosinophilic and non-eosinophilic asthma [55], type 2 (T2)-high and T2-low asthma [56,57,58,59], and asthma patients with and without aspirin-exacerbated respiratory disease (AERD) [60]. All subjects signed an informed consent form to participate in the study. All procedures and experiments associated with this research were carried out in accordance with the Declaration of Helsinki from 1975, as revised in 2008. The study was approved by the Ethics Committees of the University Hospital Centre Zagreb, Croatian Institute for Transfusion Medicine, and the University of Zagreb, School of Medicine (Project: Person-centered research of phenotypes and genotypes in asthmatic patients, Permission No: 02/21 AG).

### 2.2. Blood Collection

Sampling was performed in the morning after an overnight fast. Blood samples (8.5 mL) were collected into BD Vacutainer^®^ tubes with 1.5 mL of acid-citrate-dextrose (ACD) anticoagulant. The whole blood samples were centrifuged at 3000 rpm for 3 min at 4 °C. The obtained platelet-rich plasma was further centrifuged at 5000 rpm for 15 min at 4 °C to sediment the platelets. The pellet was then washed with saline and centrifuged again. The resulting platelet pellet was stored at −20 °C.

### 2.3. Determination of Platelet 5-HT Concentration

Platelet 5-HT concentrations were determined by the spectrofluorometric method as described previously [61]. Platelets were broken down by sonication (20 kHz, amplitude 8 × 10^−3^ mm for 30 s). Specimens of 5-HT standards, blank (water), and platelet sonicates were analyzed in duplicates. All samples were deproteinized with 1 mL of 10% ZnSO_4_ and 0.5 mL of 1 M NaOH. To prepare a fluorophore, 0.2 mL of L-cysteine (0.1%) and 1.2 mL of orthophthalaldehyde (0.05%) were added to the deproteinized samples. The 5-HT fluorescence was measured at fixed wavelengths (excitation λ = 345 nm; emission λ = 485 nm) using a Varian Spectrophotofluorometer Cary Eclipse (Agilent Technologies, USA). Platelet 5-HT levels were expressed in nmol per mg of total protein, whose concentration in platelets was quantified by Lowry et al. (1951) [62].

### 2.4. Determination of Platelet MAO-B Activity

Platelet MAO-B activity was determined using kynuramine as substrate, applying a modification of Krajl’s spectrophotoflurometric method [63], as described previously by Svob Strac et al. (2016) [64]. Standard (4-hydroxyquinoline, 4-HOQ), blank (water), and platelet sonicates were incubated with MAO-B substrate kynuramine at 37 °C. After 1 h, the reaction was stopped by adding cold 1 M NaOH. The spectrophotofluorimeter Varian Cary Eclipse, set at excitation λ = 310 nm and emission λ = 380 nm, was used to assess 4-HOQ fluorescence, a product of the oxidation of kynuramine by MAO-B. Platelet MAO-B activity was expressed in nmol of 4-HOQ/mg protein/h, with total protein concentration determined in the platelets by Lowry et al. (1951) [62].

### 2.5. DNA Extraction and Genotyping

Genomic DNA was isolated from peripheral blood by a standard salting-out procedure [65]. DNA samples were genotyped for the *MAOB* (rs1799836 and rs6651806), *HTR2A* (rs6314 and rs6313), and *HTR2C* (rs3813929 and rs518147) gene polymorphisms using TaqMan-based allele-specific polymerase chain reaction (PCR) on an ABI Prism 7000 Sequencing Detection System apparatus (Applied Biosystems, Foster City, CA, USA) according to the manufacturer’s procedures. Briefly, 20 ng of genomic DNA was PCR amplified in 96-well plates using a 10 μL reaction volume. The conditions of the PCR reaction were as follows: initially, 95 °C for 10 min, then 40 cycles at 92 °C for 15 s, and 60 °C for 60 s.

### 2.6. Statistical Analysis

Statistical analysis was performed using GraphPad Prism version 4.00 for Windows (GraphPad Software, Inc., San Diego, CA, USA). The normality of data distribution was investigated using the Kolmogorov-Smirnov test. The categorical data were presented as number (N) and percentage (%), whereas numerical (continuous) data were expressed as median with the 25th (Q1) and 75th (Q3) percentiles. Continuous variables were analyzed using the Mann-Whitney U test (to compare two groups) and the Kruskal-Wallis’s test, followed by Dunn’s multiple comparisons tests (to compare three or more groups). Two-way ANOVA has been conducted on the whole sample to assess the influence of gender and smoking and their possible interaction on the platelet MAO-B activity. Fold change (FC) was for platelet 5-HT concentrations, and platelet MAOB activity was calculated as follows: $FC=\frac{average(CASE)}{average(CONTROL)}$.

Haploview software v. 4.2 [66] was used to determine the linkage disequilibrium (LD) pairwise values (D’) between the single nucleotide polymorphisms (SNPs) in the *MAOB* (rs1799836 and rs6651806), *HTR2A* (rs6314 and rs6313), and *HTR2C* (rs3813929 and rs518147) genes, respectively. Loci were considered in LD if the D coefficient was >0.80. Genotype, allele, and haplotype frequencies were evaluated by a χ^2^-test of independence, while correlations were calculated using Spearman rank correlation. The obtained results were corrected for multiple testing, considering five comparisons of asthma phenotypes (non-severe versus severe; T2-high versus T2-low; non-allergic versus allergic; non-eosinophilic versus eosinophilic, non-AERD versus AERD), using Bonferroni correction, and, therefore, the statistical significance was defined as a *p*-value less than 0.01. Multiple linear regression analysis was performed to assess the influence of various independent variables, such as diagnosis, age, gender, BMI, smoking, and different gene polymorphisms, on the platelet 5-HT levels and MAO-B activity, as dependent variables. Moreover, receiver operating characteristic curve (ROC) analysis was performed, and the area under the curve (AUC) was determined for variables such as platelet 5-HT concentration and MAO-B activity, as well as gene polymorphisms (*MAOB* rs1799836, *MAOB* rs6651806, *HTR2A* rs6314, *HTR2A* rs6313, *HTR2C* rs3813929 and *HTR2C* rs518147). As previously described [53], the power analysis conducted using the G*Power 3 Software Version 3.1.9.2. (a free program written by Franz Faul, University of Kiel, Kiel, Germany) confirmed the appropriate sample size and statistical power of the study.

## 3. Results

Just as in our previous research [53], this study also included 120 asthma patients (41 males and 79 females) and 120 healthy control subjects (73 males and 47 females). All demographic and clinical data pertaining to the enrolled subjects have previously been described [53]. Briefly, asthma patients and healthy subjects significantly differed in their age (*p* < 0.0001), gender distribution (*p* < 0.0001) and smoking status (*p* = 0.0005), but not BMI (*p* = 0.64), with younger subjects (42 versus 58 years), predominantly males (60.83 versus 34.17%), and more current smokers (34.2 versus 8.33%) present in the control group compared to the group of asthma patients.

There was no significant correlation between platelet 5-HT concentration and age in asthma patients (*p* = 0.20, r = −0.12) or healthy individuals (*p* = 0.10, r = −0.15). Moreover, no significant correlation was observed between platelet 5-HT concentrations and BMI in asthma patients (*p* = 0.62, r = −0.04) or control subjects (*p* = 0.23, r = −0.11). When the subjects were subdivided according to gender, we observed no significant differences in platelet 5-HT concentrations between male and female healthy subjects (*p* = 0.13, U = 1437.0, Mann-Whitney test). Similarly, platelet 5-HT concentrations did not differ significantly between male and female asthma patients (*p* = 0.30, U = 1434.0, Mann-Whitney test). In addition, no significant differences were observed in platelet 5-HT concentrations between smokers and non-smokers in control (*p* = 0.80, U = 1574.0, Mann-Whitney test) or asthma (*p* = 0.34, U = 448.0, Mann-Whitney test) groups.

Platelet MAO-B activity was not significantly correlated with the age of asthma patients (*p* = 0.12, r = 0.14) or healthy subjects (*p* = 0.42, r = 0.07). Although no significant correlation was found between MAO-B activity and BMI in patients with asthma (*p* = 0.81, r = −0.02), a significant negative correlation (*p* = 0.001, r = −0.30) between BMI and platelet MAO-B activity was observed in the control group. There were significant differences in platelet MAO-B activity between males and females in the control group (*p* < 0.0001, U = 827.0, Mann-Whitney test), as well as in the group of asthma patients (*p* = 0.009, U = 1149.0, Mann-Whitney test), suggesting that female subjects in both groups have higher platelet MAO-B activity. On the other hand, platelet MAO-B activity differed significantly between smoking and non-smoking healthy subjects (*p* = 0.008, U = 1143.0, Mann-Whitney test), as well as between smoking and non-smoking asthma patients (*p* = 0.01, U = 287.0, Mann-Whitney test), suggesting lower platelet MAO-B activity in smoking subjects in both groups. Two-way ANOVA confirmed the significant influence of gender (*p* < 0.0001) and smoking (*p* < 0.0001), but not their interaction (*p* = 0.1400), on the platelet MAO-B activity.

**Figure 1:**
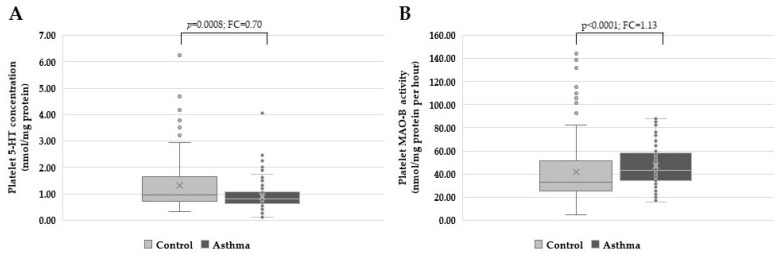
(**A**) Platelet 5-HT concentrations were significantly lower (*p* = 0.0008, Mann-Whitney test), and (**B**) platelet MAO-B activity was significantly higher (*p* < 0.0001, Mann-Whitney test) in asthma patients in comparison to control subjects. FC = fold change.

As shown in Figure 1, platelet 5-HT concentrations were significantly lower (*p* = 0.0008, U = 5388.0, Mann-Whitney test) in asthma patients (0.81 nmol/mg of protein, 0.62–1.09) when compared to healthy subjects (0.97 nmol/mg of protein, 0.72–1.65), while platelet MAO-B activity was significantly higher (*p* < 0.0001, U = 5056.0, Mann-Whitney test) in asthma patients (43.05 nmol/mg of protein/h, 34.39–58.21) in comparison to the control group (33.32 nmol/mg of protein/h, 25.71–51.26).

Multiple linear regression analysis, enrolling the whole sample (120 control subjects + 120 asthma patients), to assess the influence of not only smoking and gender but various other independent variables, such as diagnosis, age, BMI, and different gene polymorphisms, on the platelet 5-HT levels and platelet MAO-B activity, as dependent variables. Multiple linear regression revealed the diagnosis (*p* = 0.008) as a significant predictor of platelet 5-HT concentration, whereas gender (*p* = 0.00008) and BMI (*p* = 0.009) were significant predictors of platelet MAO-B activity (Supplementary Table S1).

In addition, receiver operating characteristic curve (ROC) analysis was performed, and the area under the curve (AUC) was determined for variables such as platelet 5-HT concentration and MAO-B activity, as well as gene polymorphisms (*MAOB* rs1799836, *MAOB* rs6651806, *HTR2A* rs6314, *HTR2A* rs6313, *HTR2C* rs3813929 and *HTR2C* rs518147). According to the AUC results, the platelet MAO-B activity showed the highest ability (AUC = 0.650) to distinguish between asthma patients and healthy control subjects (Supplementary Figure S1).

**Figure 2:**
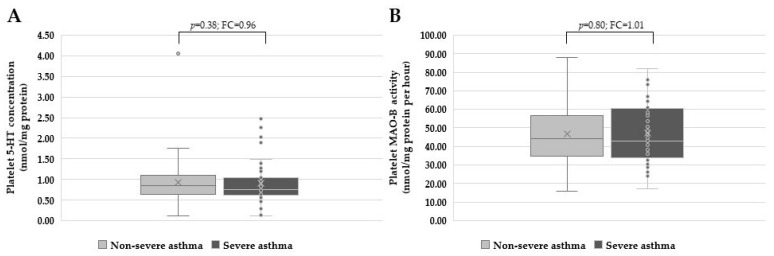
(**A**) Platelet 5-HT concentrations (*p* = 0.38, Mann-Whitney test) and (**B**) platelet MAO-B activity (*p* = 0.80, Mann-Whitney test) were not significantly different between the non-severe and severe asthma patients. FC = fold change.

Further analysis (Kruskal-Wallis and Dunn’s multiple comparison test) revealed that platelet 5-HT concentrations were significantly different (*p* = 0.004) between severe asthma patients and healthy individuals. In contrast, platelet MAO-B activity was considerably higher in non-severe (*p* = 0.006) and severe asthma patients (*p* = 0.002) than in control subjects. However, no significant differences were found between non-severe (0.85 nmol/mg of protein, 0.64–1.10) and severe (0.75 nmol/mg of protein, 0.62–1.04) asthma patients in platelet 5-HT concentration (*p* = 0.38, Mann-Whitney test), or between non-severe (44.22 nmol/mg of protein/h, 34.65–56.74) and severe asthma patients (43.00 nmol/mg of protein/h, 33.84–82.15) in platelet MAO-B activity (*p* = 0.80, Mann-Whitney test) (Figure 2).

Moreover, regression analysis that enrolled 120 patients with asthma, divided into severe and non-severe asthma patients, has been performed to check for the influence of independent parameters, including asthma severity, age, gender, BMI, smoking, and different gene polymorphisms, on the platelet 5-HT levels and platelet MAO-B activity, as dependent variables. Results demonstrated that none of the independent variables had influenced the platelet 5-HT concentration in asthma patients. However, platelet MAO-B activity was influenced by smoking (*p* = 0.010) but not by asthma severity (Supplementary Table S2). Additionally, the AUC results revealed that none of the investigated variables showed a high ability to distinguish between severe and non-severe asthma patients (Supplementary Figure S2).

**Figure 3:**
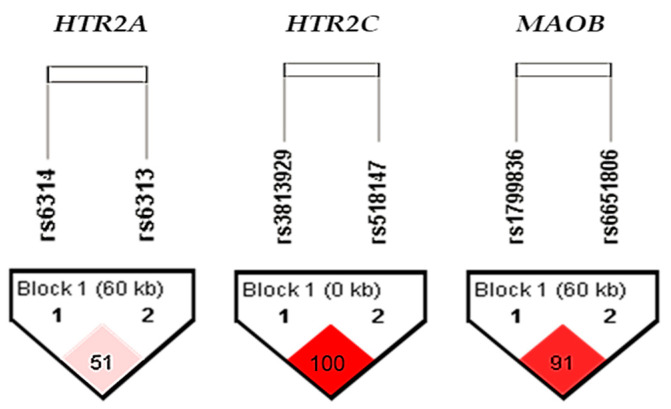
LD plot for *HTR2A, HTR2C* and *MAOB* polymorphisms in the total sample. Pairwise SNP (D′) values (×100) of linkage and haplotype blocks are shown in the diamonds. Dark-red blocks indicate SNP pairs without evidence of extensive recombination.

We further investigated the potential association of *MAOB* (rs1799836 and rs6651806), *HTR2A* (rs6314 and rs6313), and *HTR2C* (rs3813929 and rs518147) gene polymorphisms with asthma. As shown in Supplementary Table S3, no significant differences between asthma patients and healthy control subjects were observed in the frequency of the genotypes or alleles for any SNPs studied. Haplotype analysis revealed a high degree of LD for the *HTR2C* rs3813929 and rs518147 (D’ = 1), as well as for *MAOB* rs1799836 and rs6651806 (D’ = 0.91), but not for *HT2RA* rs6314 and rs6313 (D’ = 0.51) polymorphisms (Figure 3). Since a relatively low degree of LD (D’ = 0.51) has been observed for the *HTR2A* (rs6314 and rs6313) polymorphisms, indicating that these two SNPs are not likely to be transmitted together in the block, further haplotype analysis was performed only for the *HTR2C* and *MAOB* polymorphisms.

Haplotype analysis identified the three most common haplotypes of the *HTR2C* (rs3813929 and rs518147) polymorphisms, with TC haplotype (*p* = 0.04) differentially distributed between asthma patients and healthy individuals (Supplementary Table S4). However, this result was only nominally significant due to Bonferroni correction for multiple testing. As shown in Supplementary Table S4, for the *MAOB* (rs1799836 and rs6651806) polymorphisms, the four most common haplotypes were identified; however, there were no significant differences in the distribution of these haplotypes between asthma patients and control subjects.

There were no significant differences in platelet 5-HT concentration in asthma patients or healthy control subjects carrying different genotypes or alleles of the *HTR2A* (rs6314 and rs6313) or *HTR2C* (rs3813929 and rs518147) polymorphisms (Table 1).

Moreover, as shown in Table 1, there were no significant differences in platelet MAO-B activity of asthma patients carrying different genotypes or alleles of the *MAOB* (rs1799836 and rs6651806) polymorphisms. On the other hand, in the control group, nominally significant differences in platelet MAO-B activity were observed between subjects carrying different genotypes of *MAOB* rs1799836 (*p* = 0.013) and rs6651806 (*p* = 0.03) polymorphisms. However, only healthy individuals carrying the *MAOB* rs1799836 TT genotype had significantly (*p* = 0.008) lower platelet MAO-B activity than the C allele carriers (Table 1). In addition, no significant differences between non-severe and severe asthma patients were observed in the frequency of the genotypes or alleles for *HTR2A* (rs6314 and rs6313), *HTR2C* rs3813929, or *MAOB* (rs1799836 and rs6651806) polymorphisms (Supplementary Table S5). However, the frequencies of the *HTR2C* rs518147 genotypes (*p* = 0.002) and alleles (*p* = 0.05) between non-severe and severe asthma patients were significantly and nominally different, respectively. Moreover, non-severe and severe asthma patients differed significantly in the distribution of allele G carriers versus CC homozygotes (*p* = 0.004) of the *5HT2C* rs518147 polymorphism, demonstrating that the carriers of CC genotype or C allele were significantly less frequent in severe asthma patients in comparison to carriers of the G allele. Furthermore, there were no significant differences in the distribution of the *HTR2C* (rs3813929 and rs518147) and *MAOB* (rs1799836 and rs6651806) haplotypes between non-severe and severe asthma patients (Supplementary Table S6).

Finally, we examined the association between platelet 5-HT concentration and platelet MAO-B activity with different asthma phenotypes and clinical parameters (Table 2). A wide variety of clinical symptoms and phenotypes of the enrolled asthma patients has been shown in detail in our previous study by Sreter et al. (2020) [53]. A positive correlation (*p* = 0.02, r = 0.21) was observed between platelet MAO-B activity and the number of blood neutrophils. In contrast, altered platelet MAO-B activity was observed in asthma patients with pollen allergy compared to asthma patients without pollen allergy (*p* = 0.02). However, these results were not significant after correcting for multiple testing. Furthermore, there was no significant association of platelet 5-HT concentration or platelet MAO-B activity with any other examined clinical parameters of the asthma patients (Table 2).

We also analyzed the platelet 5-HT concentration and MAO-B activity in four asthma phenotypes. Table 3 shows no significant differences in platelet 5-HT concentration and platelet MAO-B activity between asthma patients with T2-high and T2-low phenotypes, eosinophilic and non-eosinophilic asthma, or with and without AERD. In addition, only a nominally significant difference (*p* = 0.047) was observed in the platelet MAO-B activity but not in the platelet 5-HT concentration between non-allergic and allergic asthma patients (Table 3).

Finally, there were no significant differences in the frequency of the genotypes, carriers, and haplotypes for any of the *HTR2A, HTR2C*, and *MAOB* gene polymorphisms studied between asthma patients with T2-high and T2-low phenotypes, non-allergic and allergic asthma, non-eosinophilic and eosinophilic asthma, or with and without AERD (data available on request).

## 4. Discussion

This research is novel in its exploration of and insights gained about the associations of platelet 5-HT concentration, platelet MAO-B activity, and the SNPs of the *HTR2A, HTR2C*, and *MAOB* genes with adult asthma in an ethnically and racially homogeneous study population (i.e., Croatian Caucasians) of asthma patients and healthy control subjects.

Conflicting findings concerning the effects of age, sex, BMI and smoking on platelet 5-HT levels and platelet MAO-B activity have been reported in the literature. For instance, some authors have shown that elderly subjects have significantly reduced 5-HT levels compared to adults and children [67]. This is opposed to the study by Kumar et al. (1998), revealing a significant positive correlation between platelet 5-HT and age [68]. Conversely, as in our case, several studies found no significant age-related associations with platelet 5-HT concentrations in healthy subjects or patients [69,70], although they did not include the asthmatic population. Moreover, our study showing no association between gender and platelet 5-HT levels in asthma patients or healthy individuals agrees with earlier studies [67,69], but in contrast to the results of higher platelet 5-HT concentration in men than in women in healthy subjects and alcoholic patients [71]. Smokers and non-smokers in our asthma and control groups had similar platelet 5-HT concentrations, which aligns with some previous reports [69,71]. On the contrary, platelet 5-HT levels were significantly affected by smoking in some other studies [61,72,73].

Certain authors have reported a positive correlation between the MAO-B activity in the brain and platelets with age [74,75,76,77,78], while others have not demonstrated any significant age-related differences in platelet MAO-B activity [79,80,81], in keeping with our findings. Our study’s multiple linear regression revealed gender and smoking as significant predictors of platelet MAO-B activity. MAO-B activity in platelets seems to depend on gender differences, as our study shows that females in both asthma and control groups had increased platelet MAO-B activity compared to their male counterparts. This is consistent with the similar findings of earlier studies showing that MAO enzyme activity was lower in men [74,79,81,82]. Concerning MAO-B, it is well established that smoking reduces the activity of this enzyme. Therefore, the decreased platelet MAO-B activity in cigarette smokers compared to non-smokers in asthma patients and healthy individuals in our study confirmed the consistent findings of many earlier studies on brain and platelet MAO-B [83,84]. In addition, Snell et al. (2002) showed that platelet MAO-B activity was significantly reduced in smokers compared to non-smokers in both males and females of different ethnic backgrounds [82]. The same association between MAO-B activity and smoking status was also reported in healthy subjects and various patients [64,80,85], although it was not investigated in patients with asthma.

Many studies suggested an association between a higher BMI and obesity with asthma [86,87]. Underlying pathophysiological mechanisms include genetic factors and obesity-related hormonal changes [88], elevated mechanical load/stress on the lungs [89], metabolic and microbiome dysregulation, as well as obesity-related low-grade inflammation [90,91]. Moreover, patients with comorbid obesity and asthma have more difficulties controlling the disease, more severe asthma symptoms and lower response to asthma treatment [91,92,93]. Two subgroups of obese asthma patients have been described: patients with an early-onset atopic asthma Th2-high, whose obesity complicates allergic asthma, and patients (usually women) with a late-onset non-atopic asthma Th2-low, whose asthma is a consequence of obesity [94]. As shown previously [53], around 23% of asthma patients in our study were obese; however, there were no differences in BMI between asthma cases and healthy controls, even when the subjects were divided into obese or non-obese individuals.

Moreover, we observed no BMI-related differences in platelet 5-HT concentration in either asthma or healthy group. This contrasts a study that demonstrated a significant negative correlation between whole blood 5-HT and BMI in healthy subjects [95] and findings showing decreased serum 5-HT levels in obese compared to non-obese individuals [96,97]. On the other hand, higher plasma 5-HT levels were reported by Young et al. (2018) in obese than in control subjects [98]. With respect to the effects of BMI or obesity on MAO-B activity in thrombocytes, our study showed a negative correlation of platelet MAO-B activity with BMI in healthy subjects but not in asthma patients. In addition, multiple linear regression suggested BMI as a significant predictor of platelet MAO-B activity in a whole sample. Ehrlich et al. (2008) found a similar result in anorexic females but not healthy women, showing a significant negative correlation between BMI and platelet MAO-B activity [99]. However, the potential role of MAO in obesity appears so far to have been generally neglected.

We demonstrated that platelet 5-HT concentration was significantly reduced in asthma patients compared to the control individuals. This finding, confirmed by the results of multiple linear regression analysis, is in line with the study by Matkar et al. (1999) that showed lower levels of platelet biogenic amines (5-HT and histamine) in asthmatic patients compared to healthy subjects in Punjabi study population [23]. In addition, our findings corroborate those of earlier studies by Malmgren et al. (1978, 1980) which reported reduced 5-HT accumulation in platelets from asthma patients in comparison to healthy individuals [21], suggesting disturbed active 5-HT transport in asthma [100]. In a later study, the same research team suggested that 5-HT uptake by platelets could be inhibited by higher amounts of 5-HT found in asthma patients’ serum and whole blood [22]. Another possible explanation for the decreased platelet 5-HT levels could be an increased platelet aggregability that is ever present in asthma patients [13]. Platelet aggregation has been shown to increase during exacerbation periods in symptomatic asthma patients and decrease during clinical improvement; therefore, increased free 5-HT levels during asthma exacerbations may be secondary to this enhanced platelet aggregation [13].

Furthermore, our study showed that platelet MAO-B activity was higher in asthma patients than healthy subjects. In fact, according to the AUC results, the platelet MAO-B activity showed the highest ability to distinguish between asthma patients and healthy control subjects. This finding is supported by Matkar et al. (1999), who demonstrated that the enzymatic levels of plasma MAO were significantly higher in asthma patients than in healthy subjects [23]. Therefore, if MAO-B is presumed to take part in the enzyme-catalyzed degradation of 5-HT in human platelets when MAO-A is absent, then an elevated MAO-B activity in asthma patients could contribute to reducing the concentration of platelet 5-HT, in comparison to healthy individuals, as observed in our study.

Moreover, our study determined that asthma severity was not associated with platelet 5-HT concentration, given that non-severe and severe asthma patients had similar platelet 5-HT levels. These findings, confirmed by the results of multiple linear regression analysis, are in contrast to the study by Lechin et al. (1996) [13], which established higher plasma 5-HT levels in symptomatic than in asymptomatic asthma patients, suggesting the association of plasma 5-HT levels with clinical severity and pulmonary function in asthma [13]. However, when comparing the healthy subjects to both non-severe and severe asthma patients, our study discovered decreased 5-HT levels only in the severe asthma patients compared to the healthy subjects. In addition, asthma severity was not associated with platelet MAO-B activity in our study, as non-severe and severe asthma patients had similar platelet MAO-B activity. Unfortunately, to our knowledge, no studies to date have been published on this topic in the literature; hence, comparisons could not be made in regard to our findings.

Our study investigated two introns *of MAOB* gene SNPs, the rs1799836 and rs6651806 polymorphisms and their haplotype, owing to their high degree of LD (D’ = 0.91). We observed no significant differences in platelet MAO-B activity of asthma patients carrying different genotypes or alleles of the *MAOB* rs1799836 and rs6651806 polymorphisms. However, healthy individuals carrying the *MAOB* rs1799836 TT genotype had significantly (*p* = 0.008) lower platelet MAO-B activity compared to the C allele carriers.

The *MAOB* rs1799836 polymorphism is believed to influence the *MAOB* intron 13 removals process and, consequently, the stability and/or translation of *MAOB* mRNA [101]. However, given that mRNA levels remain the same. At the same time, MAO-B activity varies depending on the genotype. The assumption is that there is a cis-regulatory element in LD with *MAOB* rs1799836, which alters the MAO-B protein expression and activity [102]. Lower MAO-B activity was associated with either the *MAOB* rs1799836 A allele in a small cohort of male subjects [103] or with the *MAOB* rs1799836 G allele in the postmortem human brain [102].

On the other hand, some studies have not observed a significant association between this polymorphism and MAO-B activity [80,104,105,106,107]. Our results in healthy individuals underpin the earlier findings that *MAOB* rs1799836 polymorphism influences platelet MAO-B activity. On the other hand, this association was not observed in asthma patients, probably because platelet MAO-B activity is predominantly impacted by the complex pathophysiology of asthma. Alternatively, it is possible that *MAOB* gene splicing variants could result in MAOB enzymes with different specificity to 5-HT. However, since the method for determination of platelet MAO-B activity does not use 5-HT but kynuramine as a fluorescent substrate, we could not test whether *MAOB* rs1799836 and rs6651806 polymorphisms affect the MAO-B specificity to 5-HT.

No significant differences between asthma patients and healthy control subjects or between non-severe and severe asthma patients were found in the frequency of the genotypes, alleles, or haplotypes of the *MAOB* rs1799836 and rs6651806 polymorphisms. Such associations of the *MAOB* gene polymorphisms with asthma and asthma severity have not yet been researched.

The investigated *HTR2A* and *HTR2C* gene polymorphisms in our work did not influence the platelet 5-HT levels in asthma patients or healthy individuals. Since a relatively low degree of LD (D’ = 0.51) was observed for the *HTR2A* (rs6314 and rs6313) polymorphisms, indicating that these two SNPs are not likely to be transmitted together in the block, further haplotype analysis was performed only for the *HTR2C* polymorphisms. No significant differences between asthma patients and healthy control subjects were detected in the frequency of the genotypes or alleles of *HTR2A* (rs6314 and rs6313) and *HTR2C* (rs3813929 and rs518147) polymorphisms or the frequency of *HTR2C* haplotypes. However, asthma severity was associated with the *HTR2C* rs518147 polymorphism, demonstrating that the carriers of the CC genotype or C allele were significantly less frequent in the group of severe asthma patients than carriers of the G allele. Further research is needed to elucidate the observed association.

Platelet 5-HT concentration and MAO-B activity were not significantly associated with any of the clinical parameters of asthma patients or with the asthma phenotypes (T2 high and T2 low, non-allergic and allergic, eosinophilic, and non-eosinophilic, AERD and non-AERD). In contrast, there was a significant negative correlation between free 5-HT and FEV_1_ in a study by Lechin et al. (1996) on symptomatic asthma patients [13]. However, to our knowledge, there are no published studies on the associations between platelet 5-HT levels or platelet MAO-B activity and asthma phenotypes, except allergic asthma, thus, hindering our ability to make meaningful comparisons with the literature. In patients with allergic asthma, as compared to healthy individuals, higher blood levels of 5-HT have been reported [47,108]. Notably, the 5-HT levels in these previous studies originated from serum and whole blood samples, whereas the 5-HT concentrations in our study were measured in platelets.

Moreover, we compared platelet 5-HT concentration and MAO-B activity between non-allergic and allergic asthma patients. In contrast, the earlier studies assessed 5-HT levels in allergic asthma patients only in relation to healthy subjects. In addition, our study observed no significant differences in the frequency of the genotypes, carriers, or haplotypes for any of the gene polymorphisms studied between asthma patients with T2-high and T2-low phenotypes, non-allergic and allergic asthma, non-eosinophilic and eosinophilic asthma, or those with and without AERD. However, we know that such associations of *HTR2A, HTR2C*, and *MAOB* gene polymorphisms with asthma phenotypes have not yet been investigated.

This study has some strengths and limitations. The most obvious weakness of the study is that the asthma cases and control subjects were not balanced on age, gender, or smoking status (i.e., an unmatched study design). At the same time, the sample sizes between the principal groups (i.e., asthma patients versus healthy individuals) were equal. Genetic polymorphism studies usually require very large sample sizes, but this study included relatively small numbers, chiefly due to restrictive exclusion criteria and financial constraints. Nevertheless, it was sufficiently powered with appropriate participants to observe statistically significant associations and answer the research questions. Another major strength of the study is that it focused on an ethnically homogenous population (i.e., adult Caucasian Croatians for healthy control subjects and asthma patients), which has traditionally been important for genetic studies. However, some of the outcome disparities between our and past studies may be due to the chosen demographics. Therefore, future studies should consider much larger samples with more diverse backgrounds and broader inclusion criteria to allow for the effects of comorbidities and medications, thereby better reflecting the real-world data. Furthermore, this study considered both asthma severity and several asthma phenotypes that had not yet been covered sufficiently or with consistent results in the previous literature on the serotonergic system. Therefore, it should be an area of much greater interest in future investigations on asthma.

## 5. Conclusions

In conclusion, the results of the present study indicate that asthma patients have significantly lower platelet 5-HT levels, but increased platelet MAO-B activity compared to healthy subjects. Although the reason for this remains somewhat unclear, it may nevertheless support the existence of a compensatory mechanism involving the activation of immune cells in asthma. Furthermore, our research findings imply that, neither platelet 5-HT concentration nor platelet MAO-B activity helps establish asthma severity or differentiate asthma phenotypes (i.e., allergic versus non-allergic asthma, T2-high versus T2-low asthma, non-eosinophilic versus eosinophilic asthma, or non-AERD versus AERD). Only healthy subjects, but not asthma patients, carrying the *MAOB* rs1799836 TT genotype had significantly lower platelet MAO-B activity than the C allele carriers. Therefore, platelet MAO-B activity in asthma patients is likely influenced by an interaction between genetic and environmental factors and might be predominantly affected by asthma pathophysiology. The investigated *HTR2A* and *HTR2C* gene polymorphisms did not influence the platelet 5-HT levels. No significant differences in the frequency of the genotypes, alleles, or haplotypes for any of the examined *HTR2A, HTR2C*, and *MAOB* gene polymorphisms were observed between asthma patients and healthy subjects or between patients with various asthma phenotypes. However, asthma severity was associated with the *HTR2C* rs518147 polymorphism, as evidenced by the data showing that the carriers of the CC genotype or C allele were significantly less frequent in the group of severe asthma patients in comparison to carriers of the G allele. Our findings suggest that platelet 5-HT and MAO-B activity may be helpful to peripheral blood biomarkers for asthma in general but not asthma severity or the specific phenotypes included here. However, further research is needed to clarify complex and conflicting results obtained in various studies (Table 4) and to expand upon the current knowledge about the role of peripheral 5-HT and MAO-B, as well as *MAOB, HTR2A*, and *HTR2C* gene polymorphisms in adult asthma.

## Figures and Tables

**Table 1:** Platelet 5-HT concentration and platelet MAO-B activity in control subjects and asthma patients carrying different genotypes and alleles of the *HTR2A* (rs6314 and rs6313), and *HTR2C* (rs3813929 and rs518147), as well as *MAOB* (rs1799836 and rs6651806) polymorphisms, respectively.

SNP	Platelet 5-HT Concentration (nmol/mg Protein)									
*HTR2A/C*	Genotypes				Carriers			Carriers		
rs6314	AA	AG	GG		A	GG		G	AA	
Control subjects	1.52 0.93; -	1.04 0.75; 1.70	0.91 0.69; 1.54	*p* = 0.28	1.04 0.78; 1.80	0.91 0.69; 1.54	*p* = 0.14; U = 1257.0	0.96 0.71; 1.63	1.52 0.93; -	*p* = 0.42; U = 75.0
Asthma patients	0.79 0.79; 0.7	0.88 0.69; 1.19	0.79 0.59; 1.07	*p* = 0.41	0.85 0.69; 1.18	0.79 0.59; 1.07	*p* = 0.19; U = 1079.0	0.82 0.62; 1.09	0.79 0.79; 0.79	*p* = 0.97; U = 57.0
rs6313	AA	AG	GG		A	GG		G	AA	
Control subjects	0.94 0.73; 1.6	1.01 0.74; 1.54	0.92 0.70; 1.66	*p* = 0.97	0.98 0.74; 1.61	0.92 0.70; 1.66	*p* = 0.84; U = 1522.0	0.98 0.71; 1.61	0.94 0.73; 1.68	*p* = 0.85; U = 1179.0
Asthma patients	0.81 0.62; 1.1	0.75 0.61; 1.09	0.89 0.65; 1.09	*p* = 0.39	0.76 0.62; 1.09	0.89 0.65; 1.09	*p* = 0.22; U = 1487.0	0.82 0.62; 1.09	0.81 0.62; 1.15	*p* = 0.90; U = 1022.0
rs3813929	CC	CT	TT		C	TT		T	CC	
Control subjects	1.03 0.74; 1.66	0.86 0.66; 1.22	0.92 0.78; 1.53	*p* = 0.54	0.98 0.71; 1.66	0.92 0.78; 1.53	*p* = 1.00; U = 742.0	0.90 0.70; 1.28	1.03 0.74; 1.66	*p* = 0.40; U = 1182.0
Asthma patients	0.82 0.55; 1.09	0.76 0.64; 1.12	0.76 0.56; 0.97	*p* = 0.83	0.82 0.62; 1.09	0.76 0.56; 0.97	*p* = 0.60; U = 299.0	0.76 0.64; 1.10	0.82 0.55; 1.09	*p* = 0.97; U = 1110.0
rs518147	CC	CG	GG		C	GG		G	CC	
Control subjects	1.04 0.80; 2.05	0.86 0.68; 1.13	0.99 0.65; 1.60	*p* = 0.20	0.94 0.73; 1.67	0.99 0.65; 1.60	*p* = 0.70; U = 1720.0	0.94 0.68; 1.55	1.04 0.80; 2.05	*p* = 0.12; U = 1220.0
Asthma patients	0.82 0.59; 1.08	0.76 0.66; 1.11	0.79 0.58; 1.09	*p* = 0.78	0.82 0.64; 1.09	0.79 0.58; 1.09	*p* = 0.50; U = 1661.0	0.78 0.61; 1.09	0.82 0.59; 1.08	*p* = 0.74; U = 1500.0
***MAOB***	**Platelet MAO-B activity (nmol/mg of protein per hour)**									
rs1799836	CC	CT	TT		T	CC		C	TT	
Control subjects	36.72 25.80; 59.67	40.94 31.52; 52.86	28.08 22.58; 41.41	*p* = 0.013	32.23 25.70; 51.08	36.72 25.80; 59.67	*p* = 0.40; U = 1532.0	38.85 28.61; 58.44	28.08 22.58; 41.41	*p* = 0.008 U = 1256.0
Asthma patients	41.45 31.22; 54.13	46.42 35.21; 65.73	41.45 34.66; 56.16	*p* = 0.11	43.76 35.26; 61.56	41.45 31.22; 54.13	*p* = 0.14; U = 1257.0	44.72 34.07; 58.94	41.45 34.66; 56.16	*p* = 0.59; U = 1602.5
rs6651806	AA	AC	CC		A	CC		C	AA	
Control subjects	29.81 25.07; 51.12	41.92 31.52; 61.61	30.24 21.77; 49.29	*p* = 0.03	34.13 25.73; 51.31	30.24 21.77; 49.29	*p* = 0.55; U = 1129.0	36.88 28.99; 58.69	29.81 25.07; 51.12	*p* = 0.10; U = 1438.0
Asthma patients	41.63 34.01; 59.69	45.36 35.36; 61.99	47.40 31.41; 56.31	*p* = 0.85	42.62 35.08; 59.90	47.40 31.41; 56.31	*p* = 0.69; U = 1114.0	45.76 33.72; 57.76	41.63 34.01; 59.69	*p* = 0.93; U = 1708.5

Platelet 5-HT concentration and platelet MAO-B activity are presented as median with 25th (Q1) and 75th (Q3) percentiles and compared in carriers of different genotypes (Kruskal-Wallis test) and alleles (Mann-Whitney test).

**Table 2:** The association of platelet 5-HT concentration and platelet MAO-B activity with clinical parameters of asthma patients.

Clinical Parameters	Platelet 5-HT Concentration (nmol/mg Protein)	Platelet MAO-B Activity (nmol/mg of Protein Per Hour)	
Total Serum IgE (IU/mL)	*p* = 0.52; r = 0.06	*p* = 0.66; r = 0.04	**Spearman correlation**
Blood eosinophils (×10^9^/L)	*p* = 0.63; r = 0.04	*p* = 0.39; r = 0.08	
Blood neutrophils (×10^9^/L)	*p* = 0.52; r = 0.06	*p* = 0.02; r = 0.21	
FENO (ppb)	*p* = 0.99; r = −0.001	*p* = 0.70; r = −0.04	
FEV_1_ (% of predicted value)	*p* = 0.92; r = 0.01	*p* = 0.60; r = 0.05	
FVC (% of predicted value)	*p* = 0.28; r = 0.10	*p* = 0.21; r = 0.11	
PEF (% of predicted value)	*p* = 0.20; r = −0.12	*p* = 0.73; r = −0.03	
DLCO (%)	*p* = 0.20; r = −0.12	*p* = 0.78; r = −0.03	
Duration of disease (years)	*p* = 0.99; r = −0.001	*p* = 0.63; r = 0.04	
Comorbidities (N)	*p* = 0.88; r = −0.01	*p* = 0.61; r = 0.05	
Penicillin allergy	*p* = 0.28; U = 1018.0	*p* = 0.86; U = 1159.0	**Mann-Whitney test**
Nutritive allergy	*p* = 0.69; U = 602.0	*p* = 0.43; U = 556.0	
Animal dander/feather allergy	*p* = 0.71; U = 866.5	*p* = 0.08; U = 678.0	
Dust allergy	*p* = 0.66; U = 1676.0	*p* = 0.12; U = 1469.0	
Pollen allergy	*p* = 0.30; U = 1578.0	*p* = 0.02; U = 1345.0	
Fungal/mould allergy	*p* = 0.34; U = 401.50	*p* = 0.86; U = 481.5	
Early onset of asthma (age < 12 years)	*p* = 0.99; U = 1039.0	*p* = 0.90; U = 1021.0	
History of pneumonia	*p* = 0.77; U = 997.5	*p* = 0.57; U = 957.5	
Emergency intervention (ever)	*p* = 0.82; U = 1341.0	*p* = 0.55; U = 1280.0	
Hospitalization for asthma (ever)	*p* = 0.16; U = 1529.0	*p* = 0.73; U = 1732.0	
Nasal polyps	*p* = 0.38; U = 1104.0	*p* = 0.62; U = 1164.0	
Aspirin sensitivity	*p* = 0.27; U = 472.5	*p* = 0.90; U = 580.0	
Allergen specific immunotherapy	*p* = 0.89; U = 770.5	*p* = 0.71; U = 740.0	
Oral corticosteroid therapy	*p* = 0.59; U = 1262.0	*p* = 0.09; U = 1067.0	
Biological therapy	*p* = 0.08; U = 751.5	*p* = 0.33; U = 860.5	

DLCO = diffusing capacity of the lung for carbon monoxide; FeNO = fractional exhaled nitric oxide; FEV_1_ = forced expiratory volume in one second; FVC = forced vital capacity; IgE = immunoglobulin E; PEF = peak expiratory flow; ppb = parts per billion.

**Table 3:** Platelet 5-HT concentration and platelet MAO-B activity in patients with different asthma phenotypes.

Asthma Phenotypes	Platelet 5-HT Concentration (nmol/mg Protein)		Platelet MAO-B Activity (nmol/mg of Protein Per Hour)	
T2-high (N = 94) T2-low (N = 26)	0.79 (0.62; 1.09)	*p* = 0.73; U = 1168.00 FC = 0.95	42.98 (35.30; 58.48)	*p* = 0.60; U = 1140.00 FC = 0.96
	0.91 (0.60; 1.09)		44.27 (31.81; 58.98)	
Non-allergic (N = 42) Allergic (N = 78)	0.85 (0.62; 1.18)	*p* = 0.77; U = 1585.00 FC = 1.02	37.79 (31.67; 57.17)	*p* = 0.047; U = 1278.00 FC = 1.13
	0.77 (0.62; 1.08)		44.87 (36.73; 58.60)	
Non-eosinophilic (N = 73) Eosinophilic (N = 47)	0.81 (0.62; 1.05)	*p* = 0.39; U = 1554.00 FC = 1.09	41.91 (33.63; 59.14)	*p* = 0.57; U = 1609.00 FC = 1.03
	0.86 (0.64; 1.19)		44.61 (35.32; 57.49)	
Non-AERD (N = 111) AERD (N = 9)	0.81 (0.62; 1.08)	*p* = 0.42; U = 413.50 FC = 1.05	43.15 (34.07; 58.60)	*p* = 0.71; U = 457.00 FC = 0.95
	0.82 (0.69; 1.31)		36.50 (33.93; 57.21)	

Platelet 5-HT concentration and MAO-B activity are presented as median with 25th (Q1) and 75th (Q3) percentiles and compared in patients with different asthma phenotypes using the Mann-Whitney test; AERD = aspirin-exacerbated respiratory disease. FC = fold change.

**Table 4:** The summary of findings regarding 5-HT levels, MAO-B activity, and *HTR* and *MAOB* gene variants in asthma.

Findings	Sample Type/Method	Study
↓ 5-HT platelet concentration and ↑ MAO-B platelet activity in asthma patients no association of *HTR2A, HTR2C* and *MAOB* polymorphisms with asthma	human platelets/spectrofluorometry human DNA samples/Real-Time PCR	this study
5-HT2 activation decreases airway hyperresponsiveness	BALB/c mice (BALF)/PCR, multiplex assays	[38]
↑ *HTR2A* gene expression in asthma patients vs. control group	human mononuclear cells/Real-Time PCR	[39]
↑ *5HTR2A* gene expression in asthma patients vs. control group	human PBMCs/Real-Time PCR	[45]
5-HT2 receptor activation has anti-inflammatory effects	BALB/c mice (BALF)/Real-Time PCR, ELISA	[46]
↑ *5HTR2A* gene expression in allergic asthma patients vs. control group no changes in *MAO-A* expression	human PBMCs/Real-Time PCR	[44]
↑ 5-HT levels in BALF of asthma patients ↓ 5-HT in the serum of asthma patients	mouse model and human samples (BALF, cell supernatant, plasma, serum)/enzyme immunoassay	[14]
↑ frequency of *HTR4* alleles (+142828G>A and +122769G>A) in asthma patients ↑ frequency of haplotype 1 in block 2 ↓ frequency of haplotype 4 in block 3	human DNA samples/Real-Time PCR	[41]
↑ MAO plasma activity and ↓ levels of platelet 5-HT and histamine in asthma patients	human plasma and platelets	[23]
↑ free 5-HT levels in symptomatic asthma patients vs. asymptomatic patients	human plasma/HPLC-ECD	[13,15]
no association between *HTR2* variants and bronchial asthma	human DNA samples/*MspI* restriction polymorphism	[37]
altered active 5-HT-transport in asthma patients vs. control group ↑ 5-HT plasma levels	human plasma (PPP/PRP)/^14^C- 5-HT uptake	[22,100]
↑ 5-HT during an asthma attack	human blood/fluorometry	[108]
↓ 5-HT uptake in acetylsalicylic acid-induced asthmatic patients vs. control group	human plasma (PPP/PRP)/^14^C- 5-HT uptake	[21]
↑ 5-HT serum levels in allergic subjects vs. control group	human and guinea pigs serum/spectrofluorometry	[47]

5-HT = serotonin; HPLC-ECD = high performance liquid chromatography with electrochemical detection; BALF = bronchoalveolar fluid; PPP = platelet poor plasma; PRP = platelet rich plasma; MAO = monoamine oxidase; PCR = polymerase chain reaction; PBMCs = peripheral blood mononuclear cells; ELISA = enzyme-linked immunosorbent assay.

## Data Availability

Data available on request.

## Supplementary Materials

The following supporting information can be downloaded at: https://www.mdpi.com/article/10.3390/biom13050800/s1, Table S1: The influence of various independent variables on the platelet 5-HT levels and platelet MAO-B activity in asthma patients and healthy control subjects, assessed by multiple linear regression analysis; Figure S1: Receiver operating characteristic curve (ROC) analysis was performed on the whole sample (120 control subjects + 120 asthma patients) and area under the curve (AUC) was determined for variables such as platelet 5-HT concentration and MAO-B activity, as well as gene polymorphisms (*MAOB* rs1799836, *MAOB* rs6651806, *HTR2A* rs6314, *HTR2A* rs6313, *HTR2C* rs3813929, and *HTR2C* rs518147); Table S2: The influence of various independent variables on the platelet 5-HT levels and platelet MAO-B activity in subjects with severe and non-severe asthma, assessed by multiple linear regression analysis; Figure S2: Receiver operating characteristic curve (ROC) analysis was performed on the 120 patients with asthma, divided on the severe and non-severe asthma patients and area under the curve (AUC) was determined for variables such as platelet 5-HT concentration and MAO-B activity, as well as gene polymorphisms (*MAOB* rs1799836, *MAOB* rs6651806, *HTR2A* rs6314, *HTR2A* rs6313, *HTR2C* rs3813929, and *HTR2C* rs518147); Table S3: Genotype and allele frequencies of *HTR2A, HTR2C*, and *MAOB* polymorphisms in control subjects and asthma patients; Table S4: The distribution of *HTR2C* (rs3813929 and rs518147), and *MAOB* (rs1799836 and rs6651806) haplotypes in control subjects and asthma patients; Table S5: Genotype and allele frequencies of *HTR2A, HTR2C*, and *MAOB* polymorphisms in non-severe and severe asthma patients; Table S6: The distribution of *HTR2C* (rs3813929 and rs518147), and *MAOB* (rs1799836 and rs6651806) haplotypes in non-severe and severe asthma patients.
