# Supplementary material for: Platelet Serotonin (5-HT) Concentration, Platelet Monoamine Oxidase B (MAO-B) Activity and HTR2A, HTR2C, and MAOB Gene Polymorphisms in Asthma
Source: Biomolecules. 2023 May 8;13(5):800. doi: 10.3390/biom13050800 (PMC10216077; doi:10.3390/biom13050800)
Supplement: Supplementary file 1 [file biomolecules-13-00800-s001.zip › biomolecules-2360175-supplementary.pdf]

**Table S1.** The influence of various independent variables on the platelet 5-HT levels and platelet MAO-B activity in asthma patients and healthy control subjects, assessed by multiple linear regression analysis.

| Multiple linear regression analysis | Platelet 5-HT concentration | Platelet MAO-B activity |
|---|---|---|
| **Model summary** | adj$R^2$=0.068;Δ$R^2$=0.111; F=2.585; *p*=0.004 | adj$R^2$=0.110;Δ$R^2$=0.151; F=2.585; *p*=0.00008 |
| **Diagnosis** | β=0.194; *p*=0.008 | β=0.026; *p*=0.712 |
| **Age** | β=-0.050; *p*=0.506 | β=-0.167; *p*=0.023 |
| **Gender** | β=-0.111; *p*=0.444 | β=0.266; *p*=0.00008 |
| **BMI** | β=0.052; *p*=0.193 | β=-0.175; *p*=0.009 |
| **Smoking** | β=-0.013; *p*=0.840 | β=0.040; *p*=0.520 |
| ***HTR2A rs6314*** | β=-0.138; *p*=0.045 | β=0.079; *p*=0.234 |
| ***HTR2A rs6313*** | β=0.020; *p*=0.762 | β=-0.001; *p*=0.985 |
| ***HTR2C rs3813929*** | β=-0.107; *p*=0.160 | β=-0.044; *p*=0.557 |
| ***HTR2C rs518147*** | β=-0.060; *p*=0.423 | β=-0.039; *p*=0.598 |
| ***MAOB rs1799836*** | β=0.084; *p*=0.327 | β=0.102; *p*=0.221 |
| ***MAOB rs6651806*** | β=-0.041; *p*=0.640 | β=-0.077; *p*=0.371 |

| Variables | Area under the curve (AUC) |
|---|---|
| Platelet 5-HT concentration | 0.374 |
| Platelet MAO-B activity | 0.650 |
| *MAOB* rs1799836 polymorphism | 0.494 |
| *MAOB* rs6651806 polymorphism | 0.500 |
| *HTR2A* rs6314 polymorphism | 0.542 |
| *HTR2A* rs6313 polymorphism | 0.544 |
| *HTR2C* rs3813929 polymorphism | 0.471 |
| *HTR2C* rs518147 polymorphism | 0.500 |

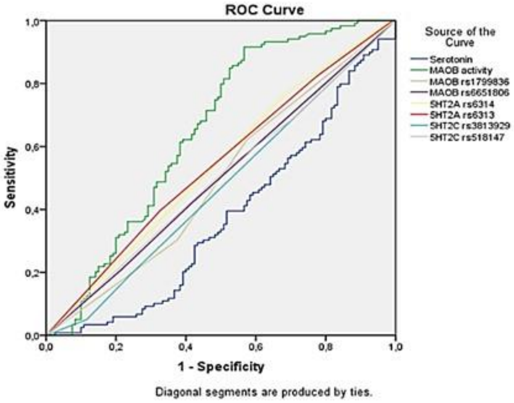


**Figure S1.** Receiver operating characteristic curve (ROC) analysis was performed on the whole sample (120 control subjects + 120 asthma patients) and area under the curve (AUC) was determined for variables such as platelet 5-HT concentration and MAO-B activity, as well as gene polymorphisms (*MAOB* rs1799836, *MAOB* rs6651806, *HTR2A* rs6314, *HTR2A* rs6313, *HTR2C* rs3813929, and *HTR2C* rs518147).

**Table S2.** The influence of various independent variables on the platelet 5-HT levels and platelet MAO-B activity in subjects with severe and non-severe asthma, assessed by multiple linear regression analysis.

| | **Platelet 5-HT concentration** | **Platelet MAO-B activity** |
|---|---|---|
| **Model summary** | adj$R^2$=-0.021;$\Delta R^2$=0.074; F=0.776; *p*=0.663 | adj$R^2$=0.092;$\Delta R^2$=0.177; F=2.090; *p*=0.027 |
| **Diagnosis** | β=0.030; *p*=0.761 | β=0.101; *p*=0.280 |
| **Age** | β=-0.017; *p*=0.876 | β=-0.212; *p*=0.046 |
| **Gender** | β=-0.005; *p*=0.960 | β=0.216; *p*=0.017 |
| **BMI** | β=0.089; *p*=0.375 | β=-0.110; *p*=0.243 |
| **Smoking** | β=-0.027; *p*=0.780 | β=0.240; *p*=0.010 |
| ***HTR2A rs6314*** | β=-0.166; *p*=0.098 | β=0.055; *p*=0.556 |
| ***HTR2A rs6313*** | β=0.021; *p*=0.830 | β=-0.030; *p*=0.744 |
| ***HTR2C rs3813929*** | β=-0.143; *p*=0.214 | β=-0.193; *p*=0.078 |
| ***HTR2C rs518147*** | β=-0.075; *p*=0.504 | β=-0.062; *p*=0.562 |
| ***MAOB rs1799836*** | β=0.047; *p*=0.708 | β=-0.055; *p*=0.642 |
| ***MAOB rs6651806*** | β=-0.235; *p*=0.078 | β=-0.014; *p*=0.908 |

| Variables | Area under the curve (AUC) |
|---|---|
| Platelet 5-HT concentration | 0.456 |
| Platelet MAO-B activity | 0.510 |
| *MAOB* rs1799836 polymorphism | 0.463 |
| *MAOB* rs6651806 polymorphism | 0.458 |
| *HTR2A* rs6314 polymorphism | 0.521 |
| *HTR2A* rs6313 polymorphism | 0.551 |
| *HTR2C* rs3813929 polymorphism | 0.484 |
| *HTR2C* rs518147 polymorphism | 0.573 |

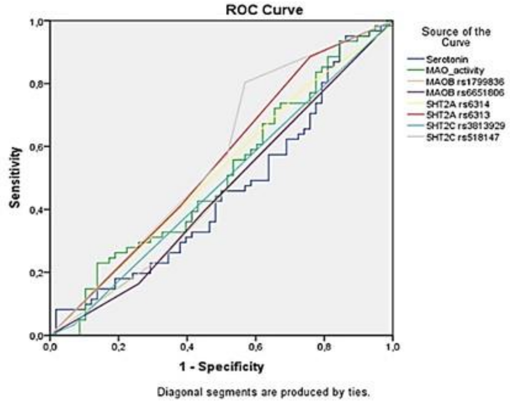


**Figure S2.** Receiver operating characteristic curve (ROC) analysis was performed on the 120 patients with asthma, divided on the severe and non-severe asthma patients and area under the curve (AUC) was determined for variables such as platelet 5-HT concentration and MAO-B activity, as well as gene polymorphisms (*MAOB* rs1799836, *MAOB* rs6651806, *HTR2A* rs6314, *HTR2A* rs6313, *HTR2C* rs3813929, and *HTR2C* rs518147).

**Table S3.** Genotype and allele frequencies of *HTR2A, HTR2C,* and *MAOB* polymorphisms in control subjects and asthma patients.

| SNP | Genotype N (%) | | | χ2-test | Allele N (%) | | χ2-test |
|---|---|---|---|---|---|---|---|
| ***HTR2A rs6314*** | **AA** | **AG** | **GG** | | **A** | **G** | |
| Control subjects | 2 (1.7) | 35 (29.2) | 82 (68.3) | *p*=0.40; | 39 (24.0) | 199 (76.0) | *p*=0.18; |
| Asthma patients | 1 (0.8) | 27 (22.5) | 92 (76.7) | χ2=2.94 | 29 (19.0) | 211 (81.0) | χ2=1.81 |
| ***HTR2A rs6313*** | **AA** | **AG** | **GG** | | **A** | **G** | |
| Control subjects | 26 (21.8) | 54 (45.4) | 39 (32.8) | *p*=0.51; | 106 (44.5) | 132 (55.5) | *p*=0.23; |
| Asthma patients | 21 (17.5) | 52 (43.3) | 47 (39.2) | χ2=2.31 | 94 (39.2) | 146 (60.8) | χ2=1.42 |
| ***HTR2C rs3813929*** | **CC/C** | **CT** | **TT/T** | | **C** | **T** | |
| Control subjects | 91 (75.8) | 15 (12.5) | 14 (11.7) | *p*=0.17; | 135 (80.8) | 32 (19.1) | *p*=0.04; |
| Asthma patients | 97 (80.8) | 17 (14.2) | 6 (5.0) | $\chi^2$=3.52 | 176 (88.4) | 23 (11.6) | $\chi^2$=4.11 |
| ***HTR2C rs518147*** | **CC/C** | **CG** | **GG/G** | | **C** | **G** | |
| Control subjects | 35 (29.2) | 21 (17.5) | 64 (53.3) | *p*=0.76; | 63 (37.7) | 104 (62.3) | *p*=0.93; |
| Asthma patients | 38 (31.7) | 17 (14.2) | 65 (54.2) | $\chi^2$=0.55 | 76 (38.2) | 123 (61.8) | $\chi^2$=0.00 |
| ***MAOB rs1799836*** | **CC/C** | **CT** | **TT/T** | | **C** | **T** | *p*=0.58; $\chi^2$=0.30 |
| Control subjects | 45 (37.5) | 24 (20.0) | 51 (42.5) | *p*=0.11; | 85 (50.9) | 82 (49.1) | |
| Asthma patients | 36 (30.0) | 38 (31.7) | 46 (38.3) | $\chi^2$=4.42 | 107 (53.8.8) | 92 (46.2) | |
| ***MAOB rs6651806*** | **AA/A** | **AC** | **CC/C** | | **A** | **C** | |
| Control subjects | 70 (58.3) | 24 (20.0) | 26 (21.7) | *p*=0.98; | 110 (65.9) | 57 (34.1) | *p*=0.64; |
| Asthma patients | 69 (57.5) | 25 (20.8) | 25 (20.8) | $\chi^2$=0.04 | 135 (68.2) | 63 (31.8) | $\chi^2$=0.22 |

**Table S4**. The distribution of *HTR2C* (rs3813929 and rs518147), and *MAOB* (rs1799836 and rs6651806) haplotypes in control subjects and asthma patients.

| Gene | Haplotype | Control subjects n (Frequency) | Asthma patients n (Frequency) | $\chi^2$-test |
|---|---|---|---|---|
| ***HTR2C*** | CG | 104 (62.3%) | 123 (61.8%), | $\chi^2$=0.01; *p*=0.93 |
| | CC | 31 (18.6%) | 53 (26.6%) | $\chi^2$=3.34; *p*=0.07 |
| | TC | 32 (19.2%) | 23 (11.6%) | $\chi^2$=4.11; *p*=0.04 |
| ***MAOB*** | TA | 84 (50.1%) | 102 (51.4%) | $\chi^2$=0.05; *p*=0.81 |
| | CC | 56 (33.4%) | 60 (30.3%) | $\chi^2$=0.39; *p*=0.53 |
| | CA | 26 (15.7%) | 32 (16.1%) | $\chi^2$=0.01; *p*=0.92 |
| | TC | 1 (0.8%) | 4 (2.2%) | $\chi^2$=1.20; *p*=0.27 |

**Table S5.** Genotype and allele frequencies of *HTR2A, HTR2C,* and *MAOB* polymorphisms in non-severe and severe asthma patients.

| SNP | Genotype (n, %) | | | χ2-test | Allele (n, %) | | χ2-test |
|---|---|---|---|---|---|---|---|
| ***HTR2A rs6314*** | **AA** | **AG** | **GG** | | **A** | **G** | |
| Non-severe asthma | 0 (0.0) | 15 (25.4) | 44 (74.6) | *p*=0.48; | 15 (12.7) | 103 (87.3) | *p*=0.77; |
| Severe asthma | 1 (1.6) | 12 (19.7) | 48 (78.7) | χ2=1.47 | 14 (11.5) | 108 (88.5) | χ2=0.09 |
| ***HTR2A rs6313*** | **AA** | **AG** | **GG** | | **A** | **G** | |
| Non-severe asthma | 14 (23.7) | 23 (39.0) | 22 (37.3) | *p*=0.20; | 51 (43.2) | 67 (56.8) | *p*=0.21; |
| Severe asthma | 7 (11.5) | 29 (47.5) | 25 (41.0) | χ2=3.28 | 43 (35.2) | 79 (64.8) | χ2=1.60 |
| ***HTR2C rs3813929*** | **CC/C** | **CT** | **TT/T** | | **C** | **T** | |
| Non-severe asthma | 47 (79.6) | 8 (13.6) | 4 (6.8) | *p*=0.67; | 83 (87.4) | 12 (12.6) | *p*=0.65; |
| Severe asthma | 50 (82.0) | 9 (14.8) | 2 (3.2) | $\chi^2$=0.78 | 93 (89.4) | 11 (10.6) | $\chi^2$=0.20 |
| ***HTR2C rs518147*** | **CC/C** | **CG** | **GG/G** | | **C** | **G** | |
| Non-severe asthma | 26 (44.07) | 3 (5.08) | 30 (50.85) | *p*=0.002; | 43 (45.3) | 52 (54.7) | *p*=0.05; |
| Severe asthma | 12 (19.67) | 14 (22.95) | 35 (57.38) | $\chi^2$=12.63 | 33 (31.7) | 71 (68.3) | $\chi^2$=3.85 |
| ***MAOB rs1799836*** | **CC/C** | **CT** | **TT/T** | | **C** | **T** | |
| Non-severe asthma | 20 (33.9) | 17 (28.8) | 22 (37.3) | *p*=0.63; | 48 (50.5) | 47 (49.5) | *p*=0.38; |
| Severe asthma | 16 (26.2) | 21 (34.4) | 24 (39.4) | $\chi^2$=0.92 | 59 (56.7) | 45 (43.3) | $\chi^2$=0.77 |
| ***MAOB rs6651806*** | **AA/A** | **AC** | **CC/C** | | **A** | **C** | |
| Non-severe asthma | 32 (55.2) | 11 (19.0) | 15 (25.8) | *p*=0.44; | 60 (63.2) | 34 (35.8) | *p*=0.21; |
| Severe asthma | 37 (60.6) | 14 (23.0) | 10 (16.4) | $\chi^2$=1.65 | 75 (72.1) | 29 (27.9) | $\chi^2$=1.56 |

**Table S6.** The distribution of *HTR2C* (rs3813929 and rs518147), and *MAOB* (rs1799836 and rs6651806) haplotypes in non-severe and severe asthma patients.

| Gene | Haplotype | Non-severe asthma n (Frequency) | Severe asthma n (Frequency) | $\chi^2$-test |
|---|---|---|---|---|
| ***HTR2C*** | CG | 52 (54.7%) | 71 (68.3%) | $\chi^2$=3.85; *p*=0.05 |
| | CC | 31 (32.6%) | 22 (21.2%) | $\chi^2$=3.35; *p*=0.07 |
| | TC | 12 (12.6%) | 11 (10.6%) | $\chi^2$=0.20; *p*=0.65 |
| ***MAOB*** | TA | 44 (46.3%) | 58 (55.8%) | $\chi^2$=1.22; *p*=0.27 |
| | CC | 31 (32.6%) | 28 (26.9%) | $\chi^2$=0.90; *p*=0.34 |
| | CA | 16 (16.8%) | 17 (16.3%) | $\chi^2$=0.00; *p*=0.98 |
| | TC | 3 (3.2%) | 1 (1.0%) | $\chi^2$=0.49; *p*=0.49 |
